# Global to USA County Scale Analysis of Weather, Urban Density, Mobility, Homestay, and Mask Use on COVID-19

**DOI:** 10.3390/ijerph17217847

**Published:** 2020-10-26

**Authors:** Sajad Jamshidi, Maryam Baniasad, Dev Niyogi

**Affiliations:** 1 Department of Agronomy-Crops, Soils and Water Sciences, Purdue University, West Lafayette, IN 47907, USA; sjamshi@purdue.edu; 2 Department of Chemistry and Biochemistry, The Ohio State University, Columbus, OH 43210, USA; baniasad.1@buckeyemail.osu.edu; 3 Department of Geological Sciences, Jackson School of Geosciences, and the Department of Civil, Architectural and Environmental Engineering, Cockrell School of Engineering, The University of Texas at Austin, Austin, TX 78712, USA

**Keywords:** COVID-19, equivalent temperature, homestay, mask use, mobility, population, urban density

## Abstract

Prior evaluations of the relationship between COVID-19 and weather indicate an inconsistent role of meteorology (weather) in the transmission rate. While some effects due to weather may exist, we found possible misconceptions and biases in the analysis that only consider the impact of meteorological variables alone without considering the urban metabolism and environment. This study highlights that COVID-19 assessments can notably benefit by incorporating factors that account for urban dynamics and environmental exposure. We evaluated the role of weather (considering equivalent temperature that combines the effect of humidity and air temperature) with particular consideration of urban density, mobility, homestay, demographic information, and mask use within communities. Our findings highlighted the importance of considering spatial and temporal scales for interpreting the weather/climate impact on the COVID-19 spread and spatiotemporal lags between the causal processes and effects. On global to regional scales, we found contradictory relationships between weather and the transmission rate, confounded by decentralized policies, weather variability, and the onset of screening for COVID-19, highlighting an unlikely impact of weather alone. At a finer spatial scale, the mobility index (with the relative importance of 34.32%) was found to be the highest contributing factor to the COVID-19 pandemic growth, followed by homestay (26.14%), population (23.86%), and urban density (13.03%). The weather by itself was identified as a noninfluential factor (relative importance < 3%). The findings highlight that the relation between COVID-19 and meteorology needs to consider scale, urban density and mobility areas to improve predictions.

## 1. Introduction

**Figure 1:**
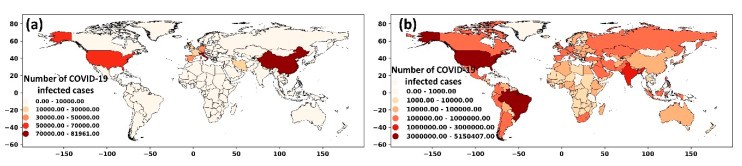
Spatial distribution of COVID-19 cases worldwide (**a**) until the end of May 2020 and (**b**) until mid-August 2020.

From late 2019, the new coronavirus (SARS-CoV-2 or COVID-19) has been swiftly spreading around the world and affected over 21 million people (with ~776,000 fatalities) worldwide as of 15 August 2020 [1]. The virus initially emerged in Wuhan, China, and the World Health Organization (WHO) was alerted on 31 December 2019 by the Chinese authorities about the pneumonia cases related to COVID-19. By the end of March 2020, the virus was pandemically propagated through Europe (e.g., Italy, Spain, France) and Asia (e.g., Turkey, Iran, India) (Figure 1a), leading to national lockdowns, quarantines, and global curbs. As of 15 August 2020, the COVID epicenter is the United States (USA), with over 5.3 million confirmed cases, followed by Brazil, India, and Russia with 3.3, 2.6, and 0.9 million cases, respectively (Figure 1b).

An increasing amount of scientific literature has focused on the environmental sensitivity of COVID-19 to modulate the risk of transmission. However, the study caveats have likely obscured plausible confounders and led to misconceptions about how weather drives COVID-19 exposure and transmission [2]. With the evolution in the virus spread, more information and dataset have become available, providing an opportunity to (re)assess the possible contributing factors to pandemic growth in populous regions such as cities. The inconsistent conclusions related to the role of weather (‘climate’), and the access to newer data, motivate the re-evaluation of the findings from the early-stage studies that assessed the link between the COVID-19 transmission and weather.

Several attempts have been undertaken to evaluate the impact of meteorological parameters on the spread of infectious diseases (e.g., influenza, SARS). For example, in northern Europe, the influenza virus’ highest activity was found during periods with low ultraviolet radiation and colder periods [2]. A higher risk of SARS transmissibility was also reported in a colder environment (16 °C to 28 °C) [3,4]. Since the beginning of the COVID-19 pandemic, researchers have linked mean air temperature [5,6] and absolute/relative humidity [7,8,9] with the pandemic growth. These studies have reported that the novel coronavirus transmission can be suppressed in warm and humid conditions analogous to a seasonal respiratory flu virus behavior [10].

The findings of these studies, however, have been based on assessments over a relatively short period (e.g., the study by Sajadi et al. [11]) or a specific geographical location (e.g., the analysis by Rosario et al. [12]). As reported in Baker et al. [13], the studies during the early-stage of the COVID-19 pandemic could be inevitably inconclusive, given the limited available data and information about the virus. Baker et al. [13] also simulated a pandemic event using a climate-dependent epidemic model and showed that climate could drive only a modest change to the pandemic size. Similarly, recent studies have reported that COVID-19 does not behave as other conventional coronaviruses based on weather and seasonality patterns. For example, Iqbal et al. [14] used wavelet transform coherence to analyze the impact of air temperature on COVID-19 spread and reported no link between temperature and transmission rate in Wuhan, China. Similarly, in other studies in Spain [15] and Iran [16], researchers reported no evidence of a relationship between COVID-19 cases and climatic parameters.

The temporal and spatial scale considered in a study can also impact the outcome and interpretation of the findings. For example, in the study by Tosepu et al. [5] in Jakarta, Indonesia, the range of vulnerable air temperature for the virus spread was reported to be between 26 °C and 28 °C. In contrast, Sajadi, Habibzadeh, Vintzileos, Shokouhi, Miralles-Wilhelm and Amoroso [11] reported the range between 5 °C and 11 °C over the USA (considering the data until March 2020), and Gupta et al. [17] reported the vulnerable air temperature between 3 °C and 17 °C over the USA (considering the data until mid-April 2020). In China, several studies [18,19] have reported the efficacy of COVID-19 transmission in cold and dry environments. In contrast, Poirier et al. [20] reported that the weather-driven parameters are not necessarily correlated with the COVID-19 outbreak when different spatial scales are considered. These inconsistencies substantiate the role of “spatiotemporal scale” and “statistical method” to interpret the impact of meteorological parameters on the COVID-19 pandemic, as noted in a review study by Briz-Redón and Serrano-Aroca [21].

Evaluating the impact of meteorological parameters on the epidemic spread of COVID-19 is challenging because of the unknown implications of several intermediate parameters on the behavior and pattern of the transmission [22]. The clinical studies have consistently reported that the most frequent and plausible coronavirus transmission routes are “droplet transmission”, “direct contact transmission” (not involving contaminated surfaces), and “indirect contact transmission” (involving contaminated surfaces) [23,24,25]. Thus, tracking the transmission routes is possible for clinical trials and not in a population-level scenario. In a real-world setting, the modes of the virus transmission are indirectly mirrored or triggered by contact-based dynamics that are, we postulate, better exemplified within an urban exposure environment. For example, a higher number of daily trips in a city with higher population density translates into a greater chance of contact among people, which increases the likelihood of the virus spread.

Specific environmental and socioeconomic components such as individual health and hygiene factors (e.g., washing hands or sanitizing, living conditions, and working environments) are difficult to track; yet, several datasets for critical factors affecting the virus transmissibility are available. Examples of critical elements and available datasets include gridded demographic data [26], mobility and foot-traffic data from Google community reports [27] or SafeGraph [28] as indicators of social and physical distancing, and mask-wearing data [29]. By account for the interplay between these factors and the virus transmission dynamics, we could reduce the uncertainty regarding the relation between weather/climate and the COVID-19 pandemic.

In this study, we evaluated the impact of a weather-driven parameter (represented by the equivalent air temperature) on COVID-19 transmissibility by considering the role of population and density (number of people per area of land), mobility and homestay metrics, and mask usage at various spatial scales from global to county levels. The findings will be of interest to policymakers, medical centers, and future risk assessment studies as communities try to develop mitigation strategies against COVID-19 and similar infectious diseases.

## 2. Materials and Methods

The study investigates the association between the equivalent air temperature (as a representative of weather) and the COVID-19 spread at different spatial scales. We first evaluated the weather-COVID-19 correlation at global and regional scales. Regions included Europe, Southeast Asia, the Eastern Mediterranean, the Americas, Africa, and the Western Pacific. Second, we considered the analysis at a national level by analyzing data for countries worldwide. Examples of the countries discussed in the study include Brazil, China, Australia, Italy, India, and the United States. Next, we performed the analysis at a finer spatial scale, considering the USA. data at state and county levels. For the USA, we further evaluated the role of weather in conjunction with urban density and population, mobility and homestay metrics, and mask usage. These factors were considered the plausible pathways that increased the likelihood of contact among people and exemplified transmission routes suggested by clinical trials. The structure of the study is provided in Supplementary Figure S1.

### 2.1. COVID-19 Data

We retrieve the daily data of COVID-19 infected cases globally (for all countries worldwide) from the World Health Organization (WHO, at https://www.who.int/) covering 1 January to 15 August 2020. At the county level over the United States, the COVID-19 data was retrieved from the Centers for Disease Control and Prevention (CDC, at https://www.cdc.gov/) and aggregated to the state and national levels corresponding to the targeted analysis. We resampled the data from daily to weekly infected cases for analyzing the transmission rate and computed the changing ratio as the percentage change between the two consecutive weeks.

### 2.2. Equivalent Temperature

Previous studies have focused on three prime weather-driven factors: air temperature, relative humidity, and absolute humidity. In this study, we used a new parameter, namely the equivalent temperature, that considers the combined effect of temperature and humidity. The equivalent temperature can be calculated from several formulations. Here we used an approximation proposed by Stull [30] and Fall et al. [31], that is particularly useful when considering gridded datasets globally.

(1) $$T_{e}=T+\frac{L_{v}}{C_{pd}}r$$

where *T* is air temperature (K), *L_v_* is the latent heat of evaporation (kJ/kg), *C_pd_* is the specific heat of dry air at constant pressure (1005.7 J/(kg·°K), and r is the mixing ratio (kg/kg). To consider the impact of air temperature at different locations, we followed the Priestley–Taylor method [32] to compute the latent heat of evaporation:

(2) $$L_{v}=2.5-0.0022\times T$$

The air temperature and mixing ratio data were retrieved from reanalysis gridded products described in the next subsection.

### 2.3. Reanalysis Dataset

To calculate the equivalent temperature globally, we used air temperature, surface pressure, and mixing ratio data from NASA’s Modern-Era Retrospective Analysis for Research and Applications, version 2 (MERRA-2) publicly available from https://disc.gsfc.nasa.gov [33]. MERRA-2 employs forecast models to combine disparate observations in a physically uniform manner and provides consistent meteorological observations as gridded datasets. While there are different reanalysis products available at a global scale, MERRA-2 delivers the relatively lowest latency (~1 month) with explicit assimilation of surface datasets and features. Hence, we used it in our analysis. To compute the equivalent air temperature over the United States, we used the North American Land Data Assimilation System (NLDAS) reanalysis data [34]. We used NLDAS data primarily due to its higher resolution (~12 km) compared to MERRA-2 (~50 km) since we needed to account for the heterogeneity and variability in the USA counties. The reanalysis data for air temperature, surface pressure, and mixing ratio were retrieved at daily (for MERRA-2) and hourly time steps (for NLDAS) and reaggregated to weekly data. The data were initially pre-processed using netCDF Operator (NCO) and Climate Data Operator (CDO) and transferred to the Python environment for further analysis.

### 2.4. Demographic Information and Urban Density

The information on the population and area for countries was retrieved from the WHO’s “Demographic and Socioeconomic Statistics” section. Since a detailed analysis was required at finer spatial scales (county-level over the USA), we used the geospatial distribution of the population over the US available from https://www.worldpop.org/ [26,35]. This data product has been generated at ~100 m spatial resolution and available in a georeferenced format from 2000 to 2020. The dataset has been evaluated and used in several studies (e.g., references [35,36,37]). The aggregated population data at 1 km spatial resolution was retrieved over the USA, and the urban areas were defined following the recommendation from the US Census Bureau. Accordingly, we described the regions with more than 2500 as the urbanized cluster and urbanized area. The urban demarcation was compared against other urban maps for three subdomains, and a good match was noted. Statistical analysis was carried out in a GIS environment to estimate the size of the urban areas. The urban density was computed as

(3) $$Urban\;Density=\frac{Population\;of\;the\;urban\;area}{Urban\;area\;\left(km^{2}\right)}$$

For each USA county, the population and density of urban areas were extracted and used to analyze COVID-19 spreading.

### 2.5. Mobility, Homestay, Mask Usage Metrics, and Statistical Analysis

For the mobility metric, we used data provided by the SafeGraph team (available upon request from https://www.safegraph.com/) and the USA Bureau of Transportation Statistics (BTS, available from https://www.bts.gov). The mobility data from SafeGraph was generated using a panel of GPS pings from anonymous cellphone devices at a spatial resolution of the census block group. For each device, the “home” was determined using the common nighttime location over a six-week time window. This information was used to determine the population that stayed at home (homestay). The number of daily trips was also collected from GPS data to compute the mobility metric. The BTS data employed a similar methodology estimating the daily travel from cellphone devices data and used a multi-level weighting method to address the geographic and temporal variations.

In this study, we aggregated the data to the county level and considered the number of trips (traveling 1–100 miles per day) as a mobility metric. We also computed the percentage change in the number of trips in 2020 compared to the same date in 2019 and considered it the mobility index (%). Considering SafeGraph and BTS criteria, the population that remained at home during the pandemic was regarded as the homestay metric. The percentage change in the number of homestay population in 2020 relative to the same date in 2019 was considered as the homestay index (%).

The mask usage data was retrieved from the New York Times GitHub repository (available at https://github.com/nytimes/covid-19-data). The data estimates the prevalence of mask-usage in the USA counties and has been generated based on many online interviews between 2nd Jul and 14th Jul 2020. The survey responses have been transferred into county-level estimates using a weighted average by age, gender, and survey respondents’ locations. The 200 nearest responses to each census tract were considered the mask-wearing estimates for that census tract and aggregated to the county level. The data set provides the percentage of the population (in each county) that used masks and is represented in five categories: NEVER, RARELY, SOMETIMES, FREQUENTLY, and ALWAYS. We considered the weighted average of the five categories with the weighting coefficient of 0.25, 0.5, 0.75, and 1, respectively, for RARELY, SOMETIMES, FREQUENTLY, and ALWAYS as the average mask-wearing population in a county. These data were only available and processed over the United States.

In addition to the spatial analysis and correlation coefficients (i.e., r^2^ or the coefficient of determination), to identify the relative importance of the contributing factors to the COVID-19, we followed the guidelines by Tonidandel and LeBreton [38] and applied multiple regression analysis. We calculated orthogonal weights (ZXk) corresponding to the number of variables (k) and regressed the dependent (number of infected cases) and independent variables (e.g., Mobility or *X_j_*) on ZXk to derive standardized regression coefficients (*β_k_* and $\lambda_{jk}$). The relative weight for each of the independent variable was calculated as: $\beta_{1}^{2}\lambda_{11}^{2}+\beta_{2}^{2}\lambda_{12}^{2}+\beta_{3}^{2}\lambda_{13}^{2}$ (Supplementary Figure S2). Using only the standardized regression weights could not accurately represent the importance of a variable as it does not appropriately partition the data variance when predictors are correlated. Using the relative importance analysis, we could expect a more accurate representation of each factor and its relative importance on the dependent variable (number of infected cases).

## 3. Results

**Figure 2:**
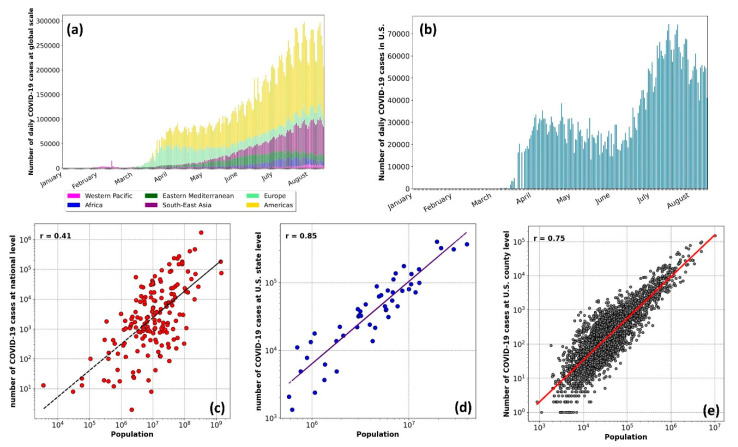
Top row: The number of daily COVID-19 cases from January 2020 for (**a**) at the global scale with the World Health Organization (WHO) regional divisions, and (**b**) in the United States Lower row: The correlation between the number of infected cases and population of the area at (**c**) the national level, (**d**) the state level over the United States, and (**e**) county level over the United States.

To provide an overview of the COVID-19 infected cases, we first presented the data at a global scale (Figure 2a) and the USA (Figure 2b). Figure 2a is categorized for different regions as per the WHO’s criterion. The highest number of cases were in the Americas, followed by Southeast Asia and Europe. During the first wave of the virus spread (until mid-May 2020), Europe and the Americas had the most significant share of the COVID-19 cases. During the second wave (from mid-May to August), the number of cases in Europe declined, while this figure increased in Southeast Asia and the Americas. Given the highest number of infections in more populous areas (e.g., the USA and Brazil in the Americas region, and India in South Asia), highlight the importance of considering the population as a factor affecting the COVID-19 transmission rate.

The strong correlation between the COVID-19 infected cases and population (r^2^ = 0.41 to 0.85) is shown at a global scale (Figure 2c), and over the USA at the state level (Figure 2d) and county level (Figure 2e). The resulting correlation highlighted the need to normalize the data relative to the population for evaluating the impacts of weather parameters. We divided the number of infected cases in a region by the regional population and considered it as the COVID-19 infected proportion.

### 3.1. Analysis at the Global Scale (Regional and National Level)

**Figure 3:**
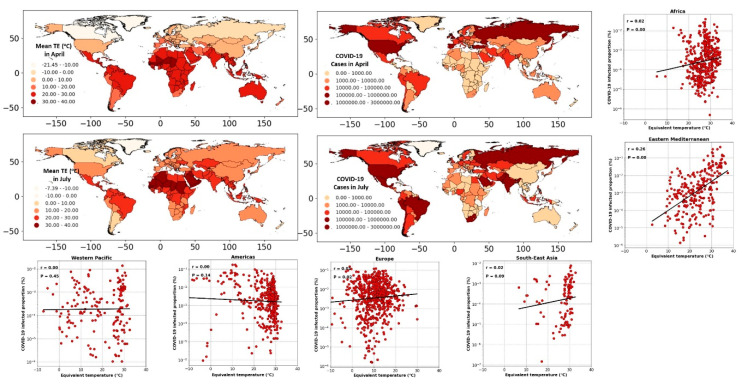
The spatial maps: global distribution of the COVID-19 cases and averaged equivalent temperature (°C) (TE) during April and July. Scatter plots: The correlation between COVID-19 infected proportion (%) and equivalent temperature (°C), considering different geographical regions from January to July 2020.

The global distribution of COVID-19 cases (aggregated over April and July 2020) and the equivalent temperature (averaged over April and July 2020) are shown in Figure 3. Comparing different regions and countries in Figure 3, we noted diverse patterns between the spreading rate and spatiotemporal distribution of equivalent temperature. In the northern hemisphere, considering the USA, the number of infected cases has risen with the increase in the equivalent temperature from April to July. Concurrently, the number of infections in Europe and Asia (e.g., in Spain, Turkey) has decreased despite higher equivalent temperatures. Similarly, in the southern hemisphere taking the examples of Australia and Brazil, we noted disparate patterns between the COVID-19 infected cases and equivalent temperature.

To better illustrate the spatiotemporal patterns mapped in Figure 3, we converted the weekly cases of COVID-19 in each country to the infected proportion. We assessed its correlation with the equivalent temperature (averaged spatially over each country). The scatter plots of Figure 3 show the resulting correlation for different geographical regions. Splitting the data into the regional segments helped reduce the bias imposed by the contrasting weather across the regions (compared to a global analysis). The resulting correlations were not statistically significant except for the Eastern Mediterranean, for which a significant but low correlation (r^2^ = 0.26, *p* < 0.05) was found. Note that in Eastern-Mediterranean countries, the onset of COVID-19 testing and collecting data coincided with warmer months of the year. In Iran, as an example of a country in the Eastern-Mediterranean region, the COVID-19 screening started in early April [39]. From April, the air temperature typically increases in the region; therefore, a biased correlation between the number of infected cases and warm weather was expected.

**Figure 4:**
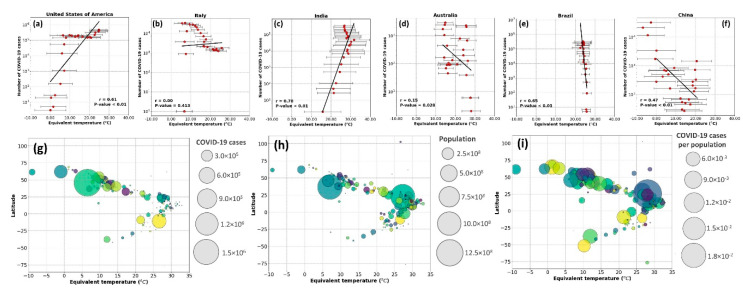
Top rows: The correlation between the weekly cumulative number of COVID-19 cases and mean weekly equivalent temperature (°C) from January to July 2020 for the (**a**) USA, (**b**) Italy, (**c**) India, (**d**) Australia, (**e**) Brazil, and (**f**) China. Lower row: visualizing the interaction between latitude, equivalent temperature, and (**g**) the number of COVID-19 cases, (**h**) population of the area, and (**i**) the proportion of infected cases normalized based on the population.

At the country level, the equivalent temperature variations were not consistent with the transmission rate. As shown in Figure 4, from January to July 2020, countries like the USA, Italy, and India have experienced a positive trend, while in China, Brazil, and Australia, the trend was negative. These assessments emphasized the importance of scale, both temporally and spatially, when the virus transmissibility is evaluated against meteorological parameters. Thus, considering the relationship between COVID-19 cases and weather-driven variables at a country level may not necessarily represent the actual behavior of COVID-19 transmission. For example, Rosario et al. [12] reported that high solar radiation and temperature could effectively suppress the spread of COVID-19 in Brazil, while Xie and Zhu [19] noted an opposite trend in China.

Some studies have linked the impact of latitude on the number of coronavirus cases, postulating that the mid-latitude countries experience more COVID-19 issues [11,40,41]. Contrary to these findings, we highlight that the latitudinal effect is invalid when we consider the country’s population. As demonstrated in Figure 4, while a larger portion of the COVID-19 cases was found in mid-latitude, we noticed that these coincide with the higher number of populations living in these areas (Figure 4h). Normalizing the data based on the population (Figure 4i), we obtained a nearly equal distribution of the COVID-19 in high, low, and mid-latitude. Therefore, the latitude was not correlated with the COVID-19 cases; instead, the population led to a higher number of infected cases in mid-latitudes.

The overall pattern achieved at the global scale is shown in Supplementary Figure S3. While the infected proportion was marginally reduced in warmer weather, the changing rate did not exhibit any consistent pattern. The absence of a trend in the changing rate of COVID-19 cases for the change in equivalent temperature showed the insensitivity of COVID-19 transmission with the increasing or decreasing temperature (Supplementary Figure S3b,c). The infected proportion was not statistically different when the equivalent temperature was between 0–10, 10–20, and 30–40 °C. Although the statistical analysis showed the highest infections in the range of −10–0 °C and the lowest in the 20–30 °C range, these findings were biased due to the significantly different frequencies of cases in the countries in these ranges (Supplementary Figure S3d).

The COVID-19 pandemic has forced the countries to adapt rapidly to new policies and procedures to stop or slow down the virus spread. Given the impact of centralized or decentralized actions on the novel coronavirus, on the one hand, and the impact of the geographical location of a region on its weather, on the other hand, it will be unlikely or too convoluted to extract the true role of weather on the pandemic growth over a large spatial scale. The analysis considering a large spatial scale can be biased due to several caveats: (i) The weather type and regime can vary distinctly across a country or a region. Assessing the averaged equivalent temperature over the whole area for correlation analysis could be subject to bias, although it provides a broad understanding. (ii) Each country and region has been subjected to different strategies and policies which were not considered in the initial assessments reported in the literature thus far. (iii) The local policies and adaptation/mitigation strategies may dynamically change during the COVID-19 pandemic depending on the magnitude of the virus spread in the area. (iv) The timing of COVID-19 testing could directly bias the result as it was exemplified in the Eastern Mediterranean region.

Overall, the inconsistent relation cross different spatial extent highlighted the limited role of weather in the COVID-19 spread. Results indicated an increase, decrease, and no change in the number of infected cases and infected proportion for a constant range of equivalent temperature. Thus, the COVID-19 spread is likely controlled by other environmental factors rather than the regional climate. Given the caveats at the global and regional analysis, we performed a more detailed analysis at a finer spatial scale (county-level over the USA) to better understand the role of climate in the pandemic growth for other possible contributing factors. Typically, the weather does not vary distinctly at the county level (compared to a regional scale) and provides a more controlled environment to assess the interplay between the contributing factors. The following sub-sections provide the analysis for the equivalent temperature, urban density, mobility, homestay, and mask-wearing for the USA counties.

### 3.2. Impact of the Urban Area and Density on COVID-19

**Figure 5:**
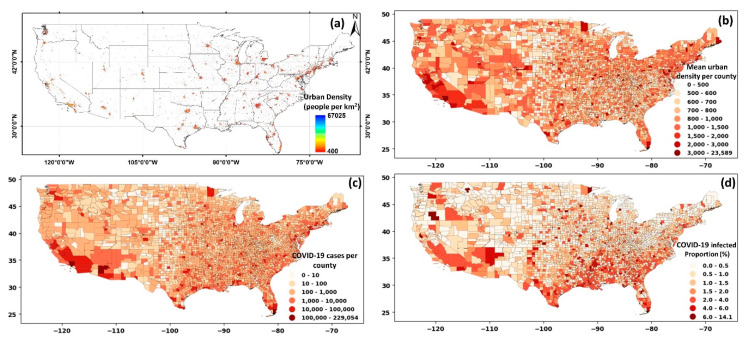
(**a**) Distribution of urban density, (**b**) averaged urban density by county, (**c**) cumulative cases on COVID-19 at the county level, (**d**) COVID-19 infected proportion at county level over the United States.

To evaluate the impact of urban area and density, we narrowed our focus over the US. The results for the urban areas and density are illustrated in Figure 5a. The mean of urban density per county was calculated, and the results are shown in Figure 5b. A visual comparison between the urban density (Figure 5b) and the total number of cases at the county level (Figure 5c) illustrated a probable correlation. The correlation between urban density and COVID-19 cases was notable in the southwest and northeast of the country. When the number of COVID-19 cases was normalized by the county-level population (Figure 5d), a different pattern emerged. The overall infected proportion was higher in southeastern and southern USA counties, with some instances in the eastern and northeastern sides.

It should be noted that the role of urban population and density differ considering their functionality and impact on COVID-19 cases. A larger population relates to a higher number of potential hosts for the virus, directly linked with the spread of the COVID-19 cases (also shown in Figure 2). A higher urban density, however, hypothetically relates to a higher chance of contact, exposure, and interactions between people, and therefore, indirectly can cause an increase in COVID-19 cases. To illustrate this difference, an example of two counties (the Suffolk and New York counties) with contrasting urban density but similar weather, imposed policies, and a relatively similar population was considered (shown in Supplementary Figure S4). 

The Suffolk and New York counties are among the most populated in the USA, with 1.48 million and 1.62 million people, respectively. However, the urban density in these two counties differs significantly, with a mean value of 32,000/km^2^ for New York county and 5800/km^2^ for Suffolk county. The substantially higher urban density in New York county compared to the Suffolk county (while the population is similar) resulted in three times higher infected cases in New York county during the early stages of the pandemic (March 2020). However, considering the data until July, a higher number of infections were found in Suffolk county, highlighting the impact of the factors beyond population. A similar pattern was also observed when all counties were analyzed. That is, other socioeconomic factors affect infection rates. The comparison here highlights that it is not just the population but the urban density that prominently contributed to the infection rates.

The number of coronavirus cases exponentially grew as urban density increased, suggesting a higher chance of COVID-19 transmission when the urban density was higher than 1400 people/km^2^ (shown in Supplementary Figure S5). After controlling the effect of the population (i.e., normalizing the number of infected cases by population), the resulting correlation was less pronounced (though statistically significant). Thus, it was not consistent with the initial hypothesis (that urban density leads to higher transmission). The temporal scale was likely the reason for the low correlation between COVID-19 infected proportion and urban density. During the early pandemic in the USA, the first cities and counties significantly affected by the virus had a dense population (e.g., the Bronx, King, Queens, and New York counties, as shown in Supplementary Figure S5c). However, as the pandemic grew, new rules and policies helped curtail the spread of the virus, making the role of urban density less apparent. The exponential increase of virus transmissibility during the early pandemic was transformed into a linear growth and showed less difference between the ranges of urban densities when all data (until July 2020) were considered (shown in Supplementary Figure S5d,e).

### 3.3. Impact of Mobility, Homestay, Mask Usage, and Weather on COVID-19

**Figure 6:**
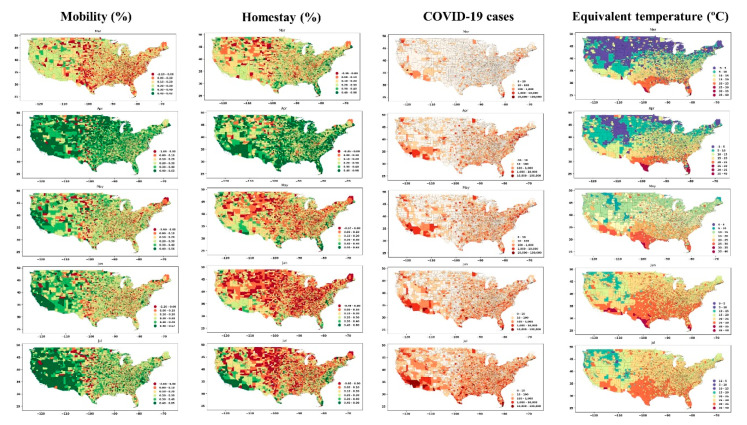
The spatial distribution of mobility and homestay change (the percent of increase or decrease in 2020 compared to 2019, with green color indicating a reduction in mobility and growth in homestay) and COVID-19 cases (third column) along with the mean equivalent temperature across the USA (rightmost column).

We evaluated the impact of mobility and homestay on the number and changing rate of COVID-19 at the USA county level. The percentage change of mobility and homestay at the county level was determined (by comparing the data in 2020 paired with a similar date in 2019), and the results are shown in Figure 6. There was no significant difference during March (early pandemic) compared to the previous year (2019). Yet, the eastern part of the country experienced more mobility (the reduction in mobility was marginally negative, i.e., red colors) compared to the west (the reduction in mobility is shown in green colors). In April, the mobility has reduced dramatically, nationwide, after that in May and July, with the imposed policies, mobility again increased. A similar pattern was observed with the change in the number of people who stayed home during the pandemic (Figure 6, homestay column). In April, the homestay was the highest, and it decreased after April. Comparing the change in mobility and homestay with the number of COVID-19, we note a feedback between these variables. From March to April, the increasing number of cases due to community spread [42] caused people to reduce mobility and increase homestay, which, in turn, slowed the rate of community spread. In May, a notable change was observed in mobility and homestay metrics, as depicted in Figure 6 (i.e., green colors shift to red from April to May). With the reopening policies, homestay percentage reduced, and mobility increased, which likely accelerated the community spread, and thus, the number of COVID-19 cases significantly increased during May in the United States. This increase coincided with the warmer season with higher temperatures and humidity (Figure 6, last column). Figure 6 provides an overview of the spatial correlation, and the changing rate of these variables was analyzed to interpret the association.

**Figure 7:**
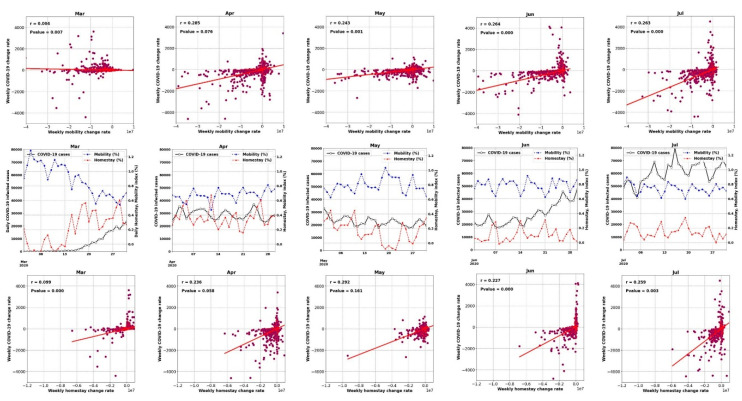
The relation between the weekly changing rate of infected cases and mobility in the form of weekly change in the number of trips (top row) and homestay in the form of weekly change in the number of home-stayed population (bottom row) over the USA counties from March to July 2020. The middle row shows the time series of daily COVID-19 infected cases aggregated for all the USA counties, and timeseries of daily mobility and homestay (%) indices averaged over the USA counties.

**Figure 8:**
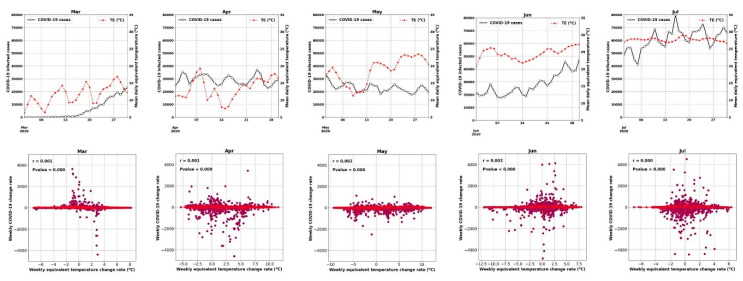
Similar to Figure 7, but for mean equivalent temperature (°C).

Figure 7 and Figure 8 show the time series of the data (for all counties) and display how the weekly percentage change of COVID-19 cases was correlated with mobility and homestay changing rates. The probability density function (PDF) and cumulative density function (CDF) associated with this analysis are shown in Supplementary Figure S6. The virus transmissibility was better correlated with mobility change and homestay. Weak correlations were noted when the COVID-19 growth rate was compared against the equivalent temperature. Considering the period from March to July, the transmissibility rate change became increasingly correlated with mobility and homestay (from r^2^ of 0.004 to 0.26, as shown in Figure 7). This trend was compatible with the general understanding of the COVID-19 spreading rate, considering a higher transmission rate with more daily trips (mobility) and less homestay. As a result, a low correlation was expected. In a population-level setting, not all the trips would lead to the virus spread. Several other factors are involved in this process, but as shown in Figure 7, the more mobility and less homestay favored the virus transmissibility. At the time of conducting this study, the imposed policies (e.g., stay-at-home directives) over the USA were mostly decentralized, issued by local authorities at the state level. Given these variable responses across different states and the complex dynamics of human mobility, it continues to be a challenge to delineate a strong association between meteorology, mobility, and homestay.

Thus, the increasing or decreasing equivalent temperature did not yield a significant correlation (r^2^ < 0.002) with the changing rate of COVID-19 cases (Figure 7). For example, during June 2020, when a 2.5 °C increase in the equivalent temperature occurred, both positive (+120 weekly infected cases) and negative (−125 weekly infected cases) changes were observed. The nearly equal distribution of these changes resulted in a neutral trend (the horizontal fit lines in Figure 7). Therefore, we could not establish any impacts of the weather (i.e., equivalent temperature) on changing the COVID-19 transmission rates.

Consistent with our results, we highlight the analysis by Kraemer et al. [43] that used near-real-time mobility data and explained the COVID-19 spread across China. Although the impact of homestay, urban density, and the weather was not included in their analysis, most of the virus spread could be explained by the travel data alone. Over the USA, Badr et al. [44] analyzed the correlation between mobility and COVID-19 cases and reported a strong correlation (r^2^ > 0.7). However, they only considered 25 counties over the USA and did not remove the population impact. The population would affect both magnitudes of the cases (as we showed) and mobility. A higher population translates into higher daily trips and mobility. From a societal impact perspective, the positive role of mobility and homestay on curbing the COVID-19 transmission has also been highlighted in Sen-Crowe et al. [45].

**Figure 9:**
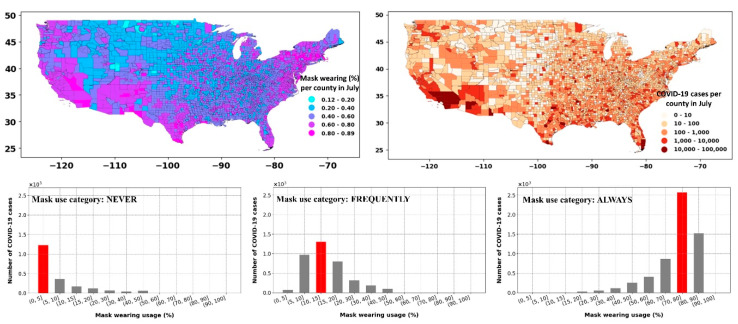
Upper row: spatial distribution of mask use across USA counties during July 2020 (according to the survey data from the *New York Times*) and COVID-19 cases during July. Lower row: commutative number of COVID-19 cases corresponding to the binned range of mask use (%). The mask usage categories shown in the lower rows figures denote the percentage of the population that “never,” “frequently,” and “always” used a mask in daily interactions.

Figure 9 shows the percent of the population in each USA county that has used a mask in daily interactions during July 2020. Comparing the mask-wearing percentage with the number of COVID-19 cases during the same period showed an “effect-and-cause” scenario. That is, in the areas with a higher infected population (i.e., southwest, coastal east), the mask-wearing percentage was also higher, suggesting that mask usage was likely dictated by the COVID-19 spread and the ensuing rules that followed. The shift in the COVID-19 cases for the percentage of people in each mask usage category highlighted the “effect-and-cause” postulation (Figure 9, lower row). For example, the counties where 70–80% of the population always used masks (considering category: ALWAYS) also corresponded to higher COVID-19 cases, while the part of the community that never used a face cover (considering category: NEVER) was in counties with low infected cases. Thus, mask use by itself was a tricky confounder that should be used with considerable caution when developing future analysis.

The limited mask-wearing data availability (only available during July) hinders our study from reflecting on the importance and explicit role of mask-wearing in the containment of the COVID-19 infection rate. However, many clinical studies confirmed the positive impact of mask-wearing on reducing the air dispersion and droplets during a human cough [30,46]. For example, Eikenberry et al. [47] have provided a conceptual model to quantify the impact of mask-wearing on the transmission rate. They reported wearing a mask by 80% of the population could reduce the projected mortality rate by 17–45% in New York, and by 24–65% in Washington state in the United States.

### 3.4. Relative Importance of the Factors Affecting COVID-19

**Figure 10:**
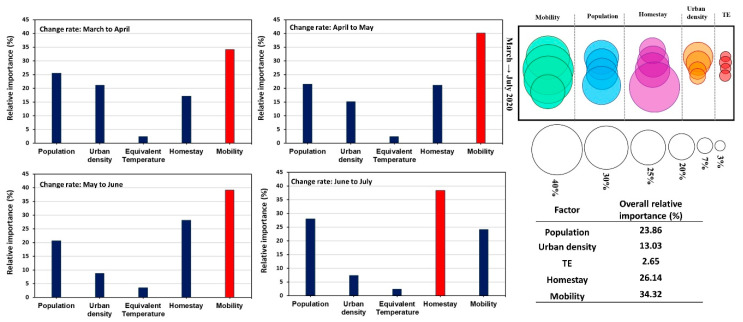
The relative importance (rescaled multiple regression weights) of different factors on the changing rate of the COVID-19 infected cases from March to July 2020 over the USA at the county level is shown as bar graphs, bubble graphs, and tables (as the overall impact for each factor).

After analyzing the impact of weather, mobility, urban density, population, homestay, and mask-wearing, separately on the COVID-19 outbreak, we conducted a multiple regression analysis [38] to evaluate the relative importance of these variables with respect to each other. The changing rate of COVID-19 cases was compared against other variables at a monthly scale, and the results are shown in Figure 10. We did not include the mask usage as the data was only available during July, and the correlation was, as discussed above, an “effect-and-cause” scenario. The multiple regression weighted coefficient was rescaled and considered as the relative importance of the variables. The resulting r^2^ of the multiple regressions varied from 0.52 to 0.57, with mobility, homestay, and population as the most important contributing factors to the COVID-19 transmissions. The importance of urban density reduced from May to July during the pandemic, which was compatible with the results previously discussed in the section related to urbanization (Section 3.2). The impact of weather (i.e., equivalent temperature) remained the least contributing factor with less than 3% of relative importance. Therefore, when evaluating the role of weather on COVID-19 transmission, it is necessary to consider factors such as population, urban density, mobility, and homestay and ensure that these effects are explicitly considered or removed from the data.

Nevertheless, our analysis showed that the impact of weather was not greater than 3%. This highlights irrespective of the weather type, without safety considerations in daily mobility, newer waves of COVID-19 can occur. We note that the analysis could be subjected to the input data’s bias as the data originated from a dynamic source, namely human activity. Additional factors, including personal health and hygiene factors, age, and re-openings, could confound the results.

## 4. Conclusions

Evaluating the impact of individual factors on the outbreak of infectious diseases, such as COVID-19, is challenging in an unsupervised environment. For most parts, this complexity stems from the dynamics of individual behavior in a complex environment (e.g., urban setting) and limitations in the data (e.g., spatial resolution, local influences). The analysis is compounded further by the spatiotemporal lags between the causal processes and effects (in this case, the exposure and the reporting). Since the start of the COVID-19 pandemic, studies have attempted to address the role of weather in regulating the COVID-19 transmission.

Our assessment indicated possible misconceptions and biases in the analysis, primarily when the study focused only on the effects of meteorological variables alone. No compelling evidence was found to include weather as a significant contributor by itself to the spread of the COVID-19. The evidence from controlled small-scale laboratory experiments on the factors affecting the transmissibility mechanism of infectious respiratory diseases (e.g., transmission through contact) highlights how these factors are manifested in real life. We found the characteristics exemplified in urban metabolism or footprint as essential considerations when COVID-19 transmission is studied. Our findings highlighted the critical role of spatial and temporal meteorological scales on interpreting the impact of environmental factors on COVID-19 spread. The function of urban density, for example, was found to be determinative considering the colossal outbreak in New York within a short time during the early pandemic (March 2020). As the COVID-19 pandemic evolved, counties with lower density showed more infections due to other dynamical factors.

At the finer scale (i.e., the USA county level), the role of urban density, mobility, homestay, and the population was evident in affecting the infection rate. These factors resulted in a low (r^2^ < 0.3) correlation at the county level in the USA, highlighting the strongly nonlinear behavior of the incorporated elements. Based on our analysis, the weather by itself was identified noninfluential factor (relative importance < 3%); therefore, when the weather is considered in a study, finer-scale data is recommended, which accounts for urban form, function, and density. Our findings can help deploy decisions and policies on the COVID-19 outbreak and restructure the role of different factors for modeling the transmission and spread of the virus that considers the urban processes, density, mobility, and population in developing an improved understanding.

## Supplementary Materials

The following are available online at https://www.mdpi.com/1660-4601/17/21/7847/s1. Figure S1: The overall structure and order of the analysis performed in the paper. Figure S2: Schematic figure of the calculations used in the relative importance analysis adapted from Tonidandel and LeBreton [38]. In the figure, we provide an example of three predictors, and the analysis (with a similar concept) was done for five predictors, including Mobility, Homestay, Weather, Population, and Urban density. Figure S3: The correlation between (a) COVID-19 infected proportion (%) and equivalent temperature (°C), (b) the change rate of COVID-19 infected proportion (day-1) and equivalent temperature (°C/day), (c) the averaged COVID-19 infected proportion for 10 °C binned range of equivalent temperature, and (d) equivalent temperature histogram. ‘ns’ stands for not significant and ‘**’ stands for statistically significant bins. Figure S4: Comparison between New York and Suffolk counties in terms of urban density and its impact on COVID-19 cases (a) during early pandemic (February to March 2020) and (b) based on all available data until August. Figure S5: The correlation between urban density and (a) COVID-19 cases, (b) COVID-19 infected proportion based on cumulative data until August 2020, (c) COVID-19 infected proportion based on data from the first two weeks of March 2020, (d) averaged COVID-19 proportion for different ranges of urban density based on data of March 2020, (e) averaged COVID-19 proportion for various degrees of urban density based on data until August 2020, and (f) histogram of the density of urban areas over the United States. Figure S6: The probability density function (PDF) and cumulative density function (CDF) of weekly infected cases (first row); mobility index (%) (second row); homestay (%) (third row); and mean equivalent temperature (°C) (fourth row) over the USA counties from March to August 2020.
