# Supplementary material for: Global to USA County Scale Analysis of Weather, Urban Density, Mobility, Homestay, and Mask Use on COVID-19
Source: Int J Environ Res Public Health. 2020 Oct 26;17(21):7847. doi: 10.3390/ijerph17217847 (PMC7663468; doi:10.3390/ijerph17217847)
Supplement: Supplementary file 1 [file ijerph-17-07847-s001.pdf]

International Journal of
Environmental Research and Public Health

MDPI

Article (supporting information)

Global to U.S. County Scale Analysis of Weather, Urban Density, Mobility, Homestay, and Mask Use on COVID-19

Sajad Jamshidi[1], Maryam Baniasad[2], Dev Niyogi[3]*

[1] Department of Agronomy- Crops, Soils, and Water Sciences, Purdue University, West Lafayette, IN 47907, USA; sjamshi@purdue.edu

[2] Department of Chemistry and Biochemistry, The Ohio State University, Columbus, OH 43210, USA; baniasad.1@buckeyemail.osu.edu

[3] Department of Geological Sciences, Jackson School of Geosciences, and the Department of Civil, Architectural, and Environmental Engineering, The University of Texas at Austin, TX 78712, USA; happy1@utexas.edu

* Correspondence: happy1@utexas.edu

Received: 6 September 2020; Accepted: 22 October 2020; Published: 26 October 2020

The supplementary materials include six figures which are provided below.

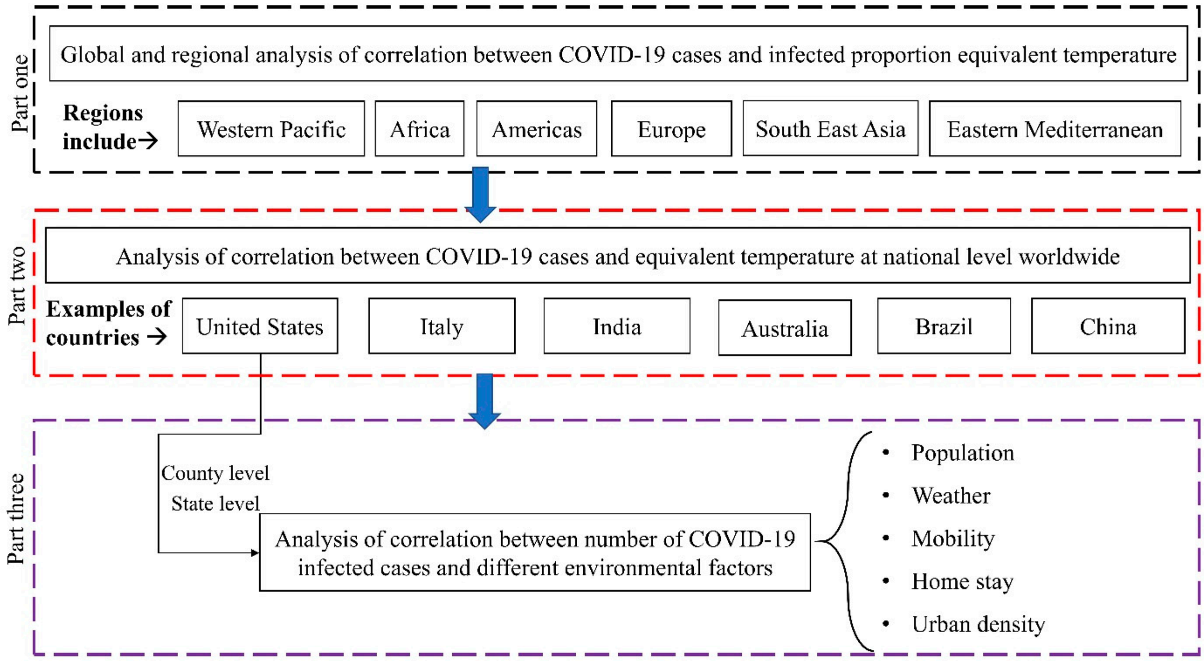

Figure S1. The overall structure and order of the analysis performed in the paper.

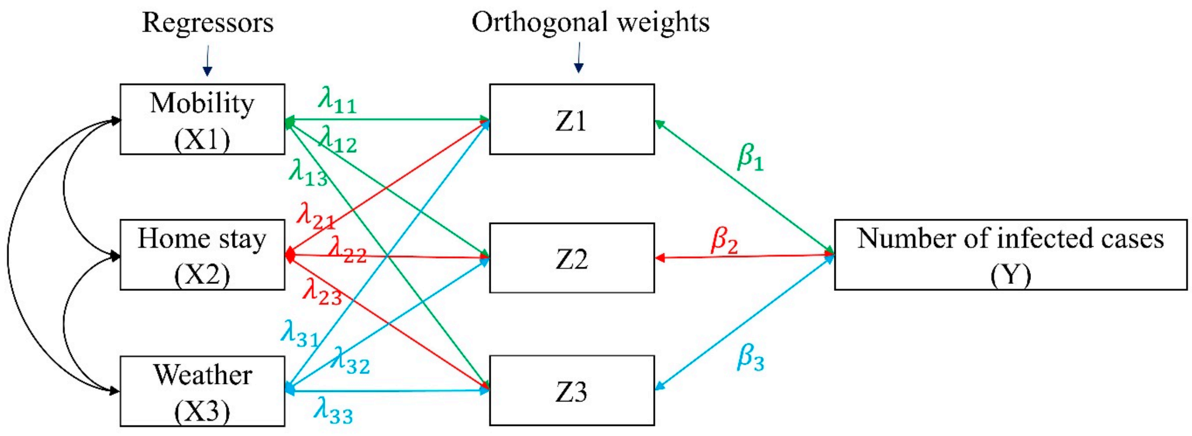

Figure S2. Schematic figure of the calculations used in the relative importance analysis adapted from Tonidandel and LeBreton (2011). In the figure, we provide an example of three predictors, and the analysis (with a similar concept) was done for five predictors, including Mobility, Homestay, Weather, Population, and Urban density.

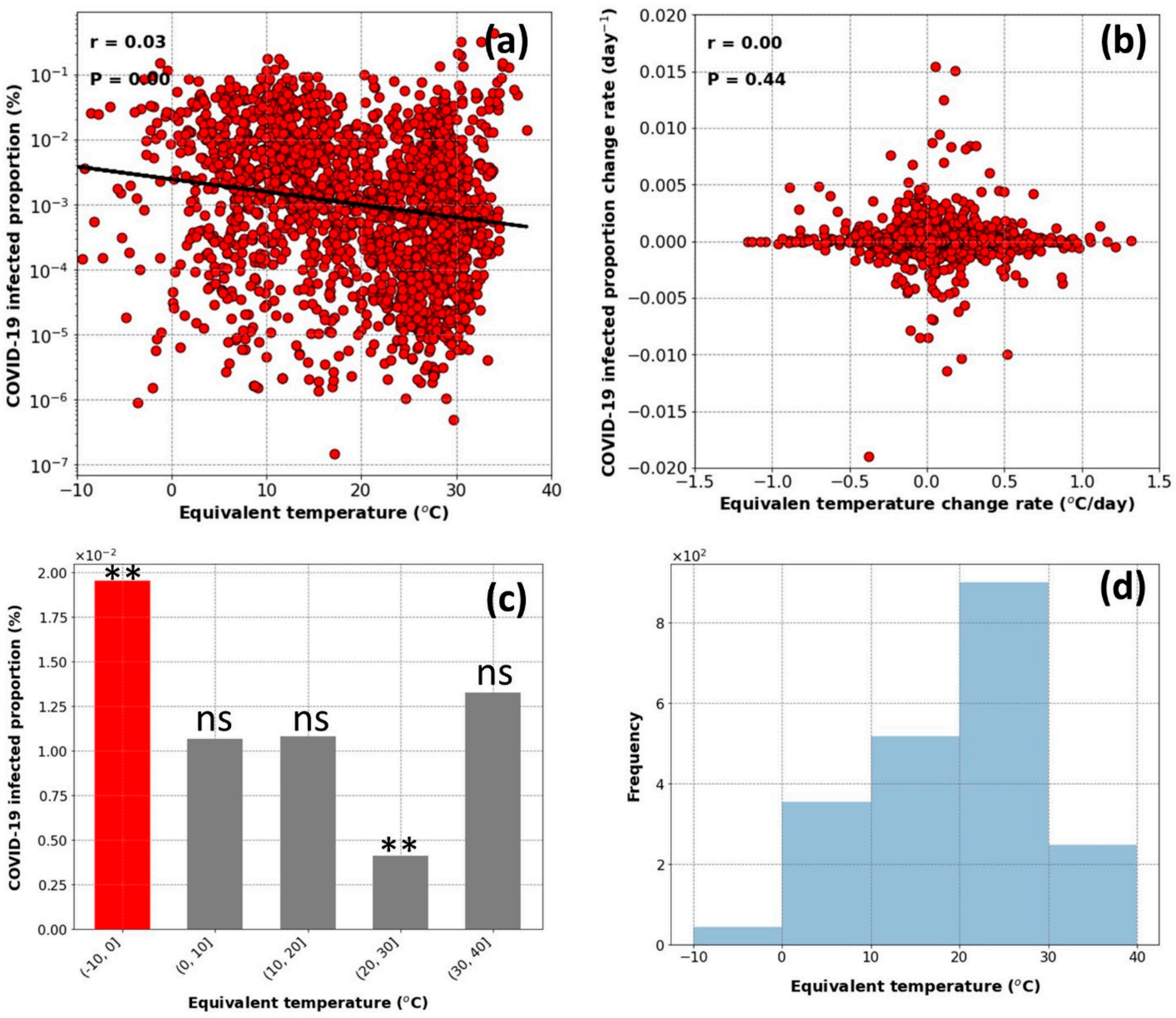

Figure S3. The correlation between a) COVID-19 infected proportion (%) and equivalent temperature (ºC), b) the change rate of COVID-19 infected proportion (day-1) and equivalent temperature (ºC /day), c) the averaged COVID-19 infected proportion for 10 ºC binned range of equivalent temperature, and d) equivalent temperature histogram. 'ns' stands for not significant and '**' stands for statistically significant bins

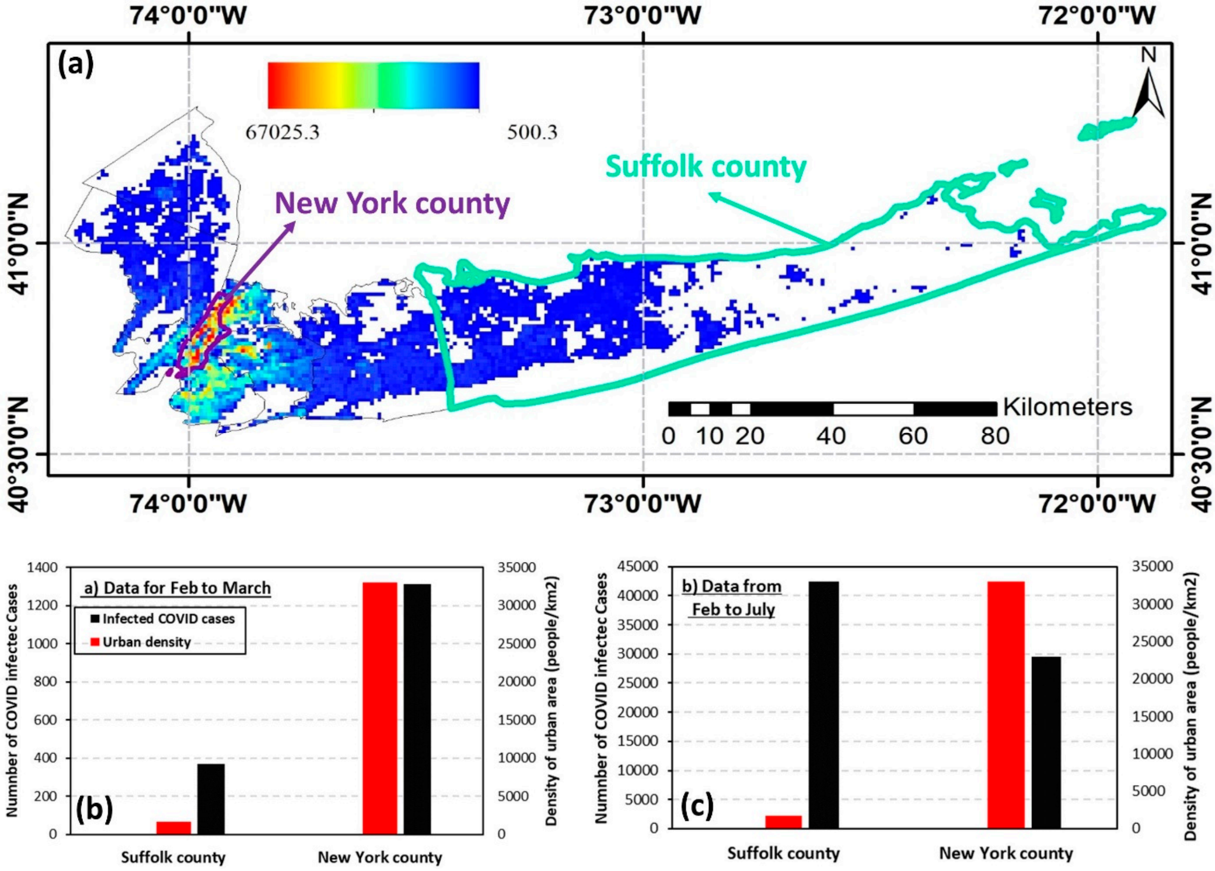

Figure S4. Comparison between New York and Suffolk counties in terms of urban density and its impact on COVID-19 cases a) during early pandemic (Feb to Mar 2020), and b) based on all available data until August 2020.

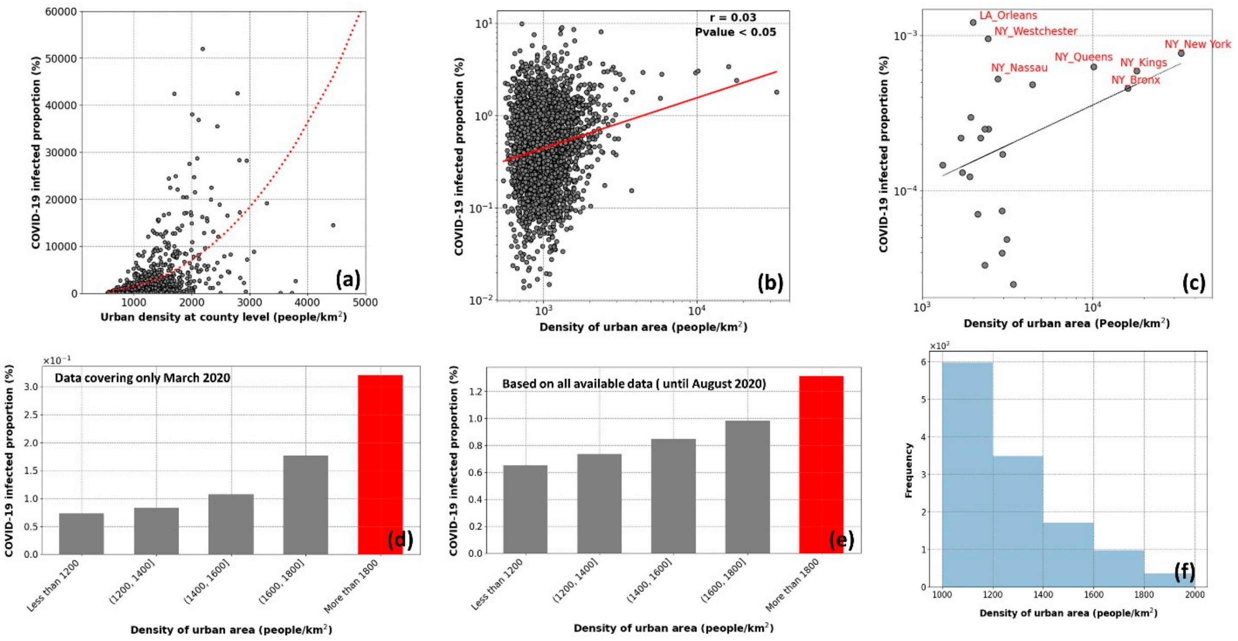

Figure S5. The correlation between urban density and a) COVID-19 cases, b) COVID-19 infected proportion based on cumulative data until August 2020, c) COVID-19 infected proportion based on data from the first two weeks of March 2020, d) averaged COVID-19 proportion for different ranges of urban density based on data of March 2020, e) averaged COVID-19 proportion for various degrees of urban density based on data until August 2020, and f) histogram of the density of urban areas over the United States.

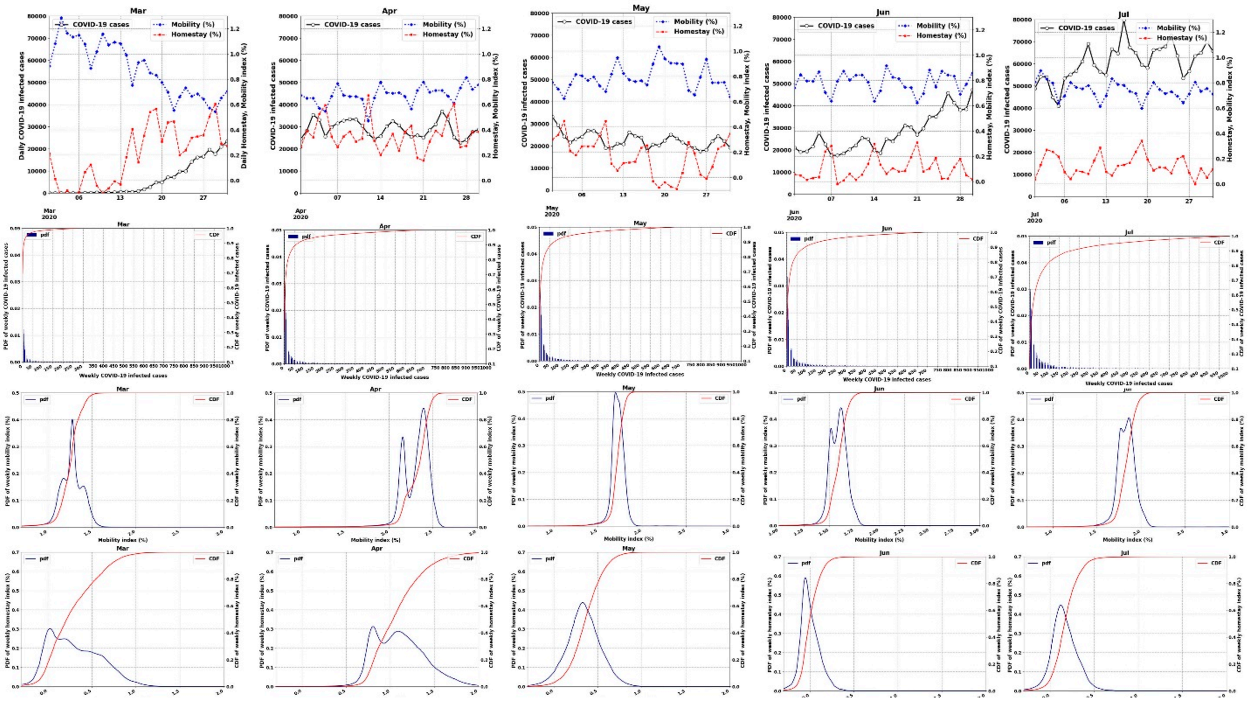

Figure S6. The probability density function (PDF) and cumulative density function (CDF) of weekly infected cases (1st row), mobility index (%) (2nd row), homestay (%) (3rd row), and mean equivalent temperature (ºC) (4th row) over the U.S. counties from March to August 2020.
